# It is Challenging to Shift the Norm: Exploring how to Anticipate and Address Microaggressions in Clinical Learning Environments

**DOI:** 10.5334/pme.1251

**Published:** 2023-12-19

**Authors:** Javeed Sukhera, Tess M. Atkinson, Justin L. Bullock

**Affiliations:** 1 Hartford Hospital and the Institute of Living, Hartford, Connecticut, USA; 2 Hartford Hospital and the Institute of Living, US; 3 University of Washington School of Medicine, Division of Nephrology, Seattle, WA, USA; 4 School of Health Professions Education, Maastricht University, Maastricht, the Netherlands

## Abstract

**Purpose:**

Increased attention to improving a culture of belonging in clinical learning environments has led to various approaches to addressing microaggressions. However, most approaches in the literature focus on responding or reacting to microaggressions with insufficient attention to building trust before microaggressions might occur. Research on microaggressions in clinical learning environments suggests anticipatory or pre-emptive conversations about microaggressions may foster greater trust. In this study, the authors explored how diverse participants perceived the experience of anticipatory conversations about potential microaggressions. Overall, the authors sought to gain a deeper understanding of how pre-emptive and anticipatory conversations may influence an organization’s approach to addressing microaggressions in clinical learning environments.

**Methods:**

The authors utilized constructivist grounded theory methodology and conducted individual qualitative interviews with 21 participants in an academic department within a larger health sciences center in the United States.

**Results:**

Findings suggest that anticipatory conversations about microaggressions were challenging due to existing norms in dynamic clinical learning and working environments. Participants shared that the idea of anticipating microaggressions elicited dissonance. Conversations about microaggressions could potentially be facilitated through leaders who role model vulnerability, organizational supports, and an individualized approach for each team member and their role within a complex hierarchical organization.

**Discussion:**

Anticipating and addressing microaggressions in clinical learning environments holds tremendous potential, however, any conversations about personal identity remain challenging in medical and healthcare environments. This study suggests that any attempts to address microaggressions requires attention to cultural norms within healthcare environments and the ways that hierarchical organizations can constrain individual agency.

## Introduction

Microaggressions are subtle comments or actions that convey hostility, invalidation or insults based on an individual’s social identity [1,2,3]. Microaggressions are common and can be both intentional and/or unintentional. Due to the harm that microaggressions can inflict on an individual’s sense of wellbeing and belonging [4], there has been a proliferation of interest in addressing microaggressions in clinical learning environments. Given the hierarchical nature of clinical learning environments, individuals who experience the harm of microaggressions require a safe and supportive environment to discuss, process, and heal from the wounds of identity-based trauma [2,12]. Existing literature on mitigating and preventing the harm of microaggressions in medical education primarily focuses on responses in-the-moment, or after the microaggression occurs [2,5,6,7,8,9,10,11], with little attention to anticipatory or preemptive measures that build a foundation of trust so that individuals feel comfortable and willing to discuss the topic of microaggressions before they might occur.

While microaggressions are rooted at an individual level, the potential preemption of microaggressions may be challenged by the hierarchical organization of clinical learning environments where discussion about an individual’s personal or social identity may challenge or threaten existing norms. Constrained Agency Theory (CAT) highlights that power in clinical organizations operates through forces that may constrain an individual’s agency to disrupt the status quo [14]. The potential for anticipatory approaches to uproot entrenched hierarchies and create an affirming and supportive learning environment, therefore requires cultivating trust and shared responsibility [13], well before any microaggression may occur. Ultimately, a deeper understanding of how preemptive approaches to microaggressions may be enacted within real-world clinical learning environments has the potential to provide insights into fostering greater inclusion and belonging for all medical learners.

An example of an empirically derived and evidence informed anticipatory approach is described by Bullock et al [15] as a Pre-brief conversation (PBC). PBC involve a pre-emptive discussion between two or more colleagues that acknowledges that microaggressions may occur and invites each individual to share their preference of how they would like others to respond. For example, when starting on a clinical rotation, the learner could be invited by the teacher to reflect on how microaggressions can occur and invite one another to share their preferential responses, should a microaggression take place. In their exploratory study of ideal bystander responses, Bullock and colleagues describe a PBC as an opportunity for staff to share their preferences regarding responses to potential microaggressions, thus offering an individualized mechanism for individuals to share their personal and cultural experiences that may influence their preferences [2,15]. Such conversations normalize vulnerability by inviting teachers and learners to share that everyone is vulnerable to the harm of identity-based microaggressions and can support each other to respond in a safe and empathic way. PBC are an example of a strategy that goes beyond training or educational interventions to foster trust and share power and agency. In this way, anticipatory approaches may be complementary to in-the-moment interventions by producing sustainable culture of support within the workplace. Yet, despite the potential strengths of PBC as an anticipatory approach regarding microaggressions, there is a paucity of literature on how such approaches may be implemented, especially in the context of medical education. Therefore, we sought to explore how a preemptive approach regarding microaggressions could be enacted within a clinical learning environment. We hoped to gain a deeper understanding of how to effectively foster belonging, trust, and safety in relation to microaggressions in clinical learning environments.

## Methods

### Approach

We utilized constructivist grounded theory (CGT) to conduct a qualitative study exploring perspectives on anticipatory approaches to microaggressions in a complex and dynamic clinical learning environment. CGT provides a useful approach when researchers seek to better understand a social process that is not well-explained by established research [16]. CGT aligns with an iterative and theory-informed approach that builds upon existing literature on challenging and addressing microaggressions in medical education. It is an approach that offers openness and flexibility, as researchers engage in constant comparative analysis to code and analyze data that informs a theoretical framework that can be built upon through future research.

### Setting and Recruitment

Participants included diverse professions and roles in a Department of Psychiatry at an Academic Health Sciences Center in the Northeastern United States of America. Clinical learning environments such as acute psychiatric units often elicit challenging and complex conversations about microaggressions due to circumstances where patients may have altered mental status or may be in acute situational crises. We felt this would provide a real-world context that could illuminate details on how to successfully address microaggressions. Participants included all types of staff ranging from those working in inpatient and ambulatory settings, as well as individuals working in various roles relating to clinical care, research, and education. Recruitment was initiated through an institution-wide email that disseminated information about the study, inclusion criteria and what participation involves. Eligible candidates were encouraged to disclose their interest in participating, and interviews were scheduled on an individual basis. Ethics approval was obtained from the organization’s research ethics board.

### Data Collection, Analysis, and Reflexivity

We sought to include a diverse set of participants with varying roles and with varying positions within the organization’s hierarchy. Our approach was consistent with CGT’s emphasis on both a purposive and theoretical sample. Semi-structured qualitative individual interviews were conducted both in person and online (via videoconference). Each interview began with answering questions and obtaining consent, followed by an introduction to key terms, and exploratory questions about the implementation of PBC in the participant’s unique context. In our initial interviews which included definitions and open questions, we found that our initial participants struggled with understanding the concept of a PBC and therefore we adapted our approach to the interview by demonstrating a role-play of a PBC and inviting the participant to practice the pre-brief with the interviewer. Consistent with CGT, we iteratively adapted our interview guide as we engaged in constant comparative analysis.

Our team was composed of JS, first author, who is a racially minoritized male, psychiatrist, and scientist in health professions education. TA who is a White female and master’s level research associate in social sciences, and JLB who is a Black, gay, nephrology fellow with lived experience of mental illness but who has no prior affiliation with the institution from which individuals participated. Our team addressed reflexivity through Walsh’s [17] reflexivity typologies; personal, interpersonal, methodological, contextual, and collaborative. Dynamics between the interviewer, TA, and each participant varied based on the individual. Some tended to be aware of the power dynamic of the interviewer and the information the participant would provide, often asking if any of this information would get back to a supervisor. In that case, participants were re-assured that all information collected was confidential, and that they could withdraw at any time at no penalty to them. JS and TA were involved in line-by-line coding of all transcripts and JLB participated in axial coding and discussion. The team resolved differences through consensus. Data were gathered until the team felt there was a sufficient understanding of the initial research question to build theory from our findings [18]. Participants were provided a brief narrative summary of the findings to facilitate member checking.

Overall, 21 individuals participated in our study. We invited participants to self-describe their identity using their own words. 19 identified as women and 2 as men. There were 4 psychologists, 4 psychiatric technicians, 3 psychiatrists, 3 administrative staff, 3 social workers, 3 research assistants, and 1 nurse. Three participants described themselves as Black or African American, 12 as White, 3 as Mixed Race, and 2 as Latina. 1 did not choose any racial or ethnic identifiers.

## Results

Having an anticipatory conversation about potential microaggressions was perceived as challenging due to norms within clinical learning environments that may constrain an individual’s agency to contest hierarchical norms. Discussing the possibility of microaggressions created dissonance within a culture where individuals felt they were not permitted to bring their full selves and social identities into their workplace. There was also a tension between having such conversations and participants’ perception of available time and their organization’s prioritization of other initiatives. Participants suggested that an understanding of power asymmetries was necessary for meaningful pre-emptive conversations about microaggressions to occur. Participants suggested that leadership support, structural changes, space to practice, prompts, and role modeling, were potential strategies to facilitate implementation.

### Perceived barriers

#### Grappling with Dissonance: “It doesn’t feel … natural”

Throughout our study, several participants noted that the concept of an anticipatory conversation about microaggressions did not feel natural or normal for them due to a workplace culture where individuals were expected to set firm boundaries between their personal and professional selves, and where issues of bias and prejudice have been pervasive for a long time. Some participants struggled to understand the concept and repeatedly shared how the idea made them “*confused*” S5, while others stated that, “*I think it sounds good in theory…I’m trying to picture like my work environment, and it doesn’t feel… natural*.” S14. When asked why PBC did not feel natural, participants pointed to a workplace culture that did not embrace or celebrate vulnerability. One participant shared that in the context of a healthcare working environment, it is often difficult to admit that they might be sensitive to microaggressions placing PBC in the larger context of help seeking, “*…just the nature asking for help is difficult in so many ways for so many reasons*,” S12 and another stated that,

“Because I think there’s a lot of different reasons that people may not, you know, bring their full selves to work. Whether it’s like exhaustion, wanting to have those boundaries, being uncomfortable, you know, being newer in a place where everybody already seems to know each other.” S21

Anticipatory conversations therefore provoked considerable dissonance and discomfort that was linked to a fear of judgment or fear of potential repercussions. One participant shared, “*It feels awkward. It would have to be something I would have to get used to doing…*” S13 and several others described fear. One described the fear that was intrinsic to revealing aspects of their social identity that were otherwise invisible stating that explicitly acknowledging their identities, “*was uncomfortable for me anyways because when I’m coming to work and I’m thinking to myself, this (my identity) is the way you guys see me*.” S18, and another described how anticipatory conversations required trust and that this was not always apparent in all their workplace relationships,

“So there are like there are some people who are authentic and I could easily go to and say hey, you know, and have this pre briefing thing or this conversation and there are just some that you would never even… because you know, you can’t trust them” S16

Another minoritized participant noted that they had experienced significant harm through their previous experiences in the organization, noting they “*don’t feel comfortable*” because they fear the organizational response to their disclosure is “*going to hurt me even more when I’m in such a hurt state*.” S19.

#### Tensions with Efficiency Culture

Participants also shared that initiating or participating in anticipatory conversations felt somewhat awkward within a workplace culture that prioritized efficiency. One participant noted that such conversations feel like they would require “*extra time*” and that barriers will persist “*until there is a structure in place where this is a conversation that everybody is having*.” S17. Another stated, that such conversations feel challenging in a culture where some employees working in direct clinical areas are simply focused on “*getting our paycheck*.” S16. Similarly, challenges to implementation included a perception that pre-emptive conversations will not lead to change because participants felt jaded that most healthcare organizations were not interested in prioritizing equity or inclusion over efficiency stating,

“How willing I would be to engage…would depend on like how I perceive that the organization is going to react like is. Is this going to do anything? Like is this going to actually change anything? If I saw there was a real effort put in and people are making changes and wanting to address things and that would make me more likely to speak up as opposed to like, If my experience has been that whenever I speak up, nothing happens, and why would I bother?” S14

#### Influence of Power and Organizational Hierarchy

Further analysis of potential barriers suggested that anticipatory conversations were challenging due to the pervasive power imbalances within organization that can influence an individual’s sense of safety in bringing their full self to the workplace. Participants noted that profession-based power differentials would impact how such conversations would take place. For example, anticipatory conversations would be different for professions that could be perceived more dominant in the workplace hierarchy such as medicine, compared to environmental service workers or research assistants. This complexity was highlighted by a participant who shared,

“I guess there might be… power imbalances are often talked about, at least in our workplace here just with the clinician there’s varying levels…I would say a barrier for at least the individuals who might not have a doctorate degree would maybe just be that power imbalance of going to talk to a clinician about that because they might be nervous that they should have handled the situation better.” S6

### Shifting the norm: Approaches to facilitate agency within a hierarchical organization

Participants described several potential ways that anticipatory conversations could be facilitated. Above all, there was a sense that facilitating implementation required shifting from what was perceived as normal within existing healthcare culture to a different way of seeing and framing conversations about microaggressions. The most salient factors that could facilitate implementation included leadership involvement and role modeling. Participants felt sensitized to the role of the leader in a hierarchical system and were particularly concerned that leaders must take responsibility for implementing change related to equity and inclusion rather than delegating this responsibility to others. One reflected on their perception that leadership role modeling makes a difference by stating,

“Let’s have meetings about it and you don’t show up then what does that tell me… that you don’t care that it’s not important to you. So why should it be important to me? And if it is not important to the higher-ups then not means that everybody who you delegate under don’t take it seriously and it’s not important to them… if you don’t take it seriously…if you don’t feel like it’s important for you to show up and talk about it and be there then why would anybody else think is important too.” S4

Others suggested that leaders and managers should be explicit if conversations about microaggressions are expected and one participant stated that organization leadership and faculty have to create a sense of safety and support, noting, “*Making it normalized, starting with the people who were perceived with the most power … so that the people who feel less inclined to talk about discrimination in the workplace would feel more comfortable to bring that up*.” S6

Participants also emphasized the importance of structural supports and meaningful integration of anticipatory conversations into their standard workplace practices. To promote culture change, such conversations must be explicitly included and scheduled “*in all meetings to help with consistency*.” S4 One participant stated that scheduling PBC can help individuals “*take it seriously and make time to have that conversation*,” S5. Another stated *We just have to put it out there…it is integrated into the format of how we interact as colleagues*.” S3

Another suggested that interprofessional team meetings and rounds should incorporate PBC to promote curiosity and “*continually seeing the benefits*,” noting that “*…by having richer conversations*” over time, people will become “*used to being vulnerable*.” S1 Overall, creating structures and consistency was also complemented by a sense of safety and support.

Role modeling and space to practice were also important enablers. For example, for individuals to share the support they need from others and support their colleagues, they need to be able to be practice such conversations to feel comfortable. One participant stated,

“I think that modeling is helpful…maybe giving the person an idea of things they might want to talk about…Do you feel comfortable talking about your religious background, your gender? … Do you feel comfortable talking about your politics?.” S13

They went on to suggest that such conversations could be more effectively implemented in team members had space to practice with “*strangers*” before implementing within an environment where people had pre-existing relationships. Another suggested that it would be helpful to “*illustrate*” a “*good conversation*” that was “*specific*” about their unique identities. S14

## Discussion

Through exploring the barriers and enablers of implementing anticipatory approaches, we learned that a pre-emptive approach to address microaggressions was fundamentally challenged by sociocultural norms in hierarchical organizations. Barriers and challenges predominate our findings, while facilitators were more difficult to elicit. Participants consistently described how they felt uncomfortable and afraid about the idea of explicitly acknowledging their personal identities in a professional context. Participants also noted that successful implementation required individuals with perceived power (such as senior organizational leaders) who role modeled vulnerability and afforded individuals in the workplace to individuate their approach to preventive conversations.

The role of emotional vulnerability is consistent with previous research on microaggressions in medical education. It is well established that medical faculty experience discomfort when discussing racism, discrimination, and microaggressions [19,20,21,22]. Faculty who had experienced microaggressions in their own personal and professional lives were sensitized to notice similar microaggressions, while some felt that they had to minimize these events. Emotional vulnerability also arose from multiple sources in such circumstances [19]. Our study provides an opportunity to learn more details about potential barriers, while shedding light on how address such barriers and advance more culturally sensitive and psychologically safe learning and working environments.

Constrained Agency Theory suggests that hierarchical organizations may constrain individual agency for counter normative practices such as preventive conversations to occur [14]. In a medical education context, the work of exercising agency can be compounded by a system that promotes conformity and therefore resists agency [23]. Yet, our decision to explore how a pre-emptive conversation such as PBC is enacted within clinical learning environments sought to disrupt such norms and leverage ingredients such as social capital, self-knowledge, and mentorship to foster agency [23]. Our findings suggest that promoting agency remains challenging and provides suggestions on how to bolster agency while advancing work towards inclusion and belonging. Participants believed that agency was promoted by organizational leaders who role-modeled such conversations and were explicit and transparent about organizational supports and actions to address diversity, equity, and inclusion.

### Exploring preventive conversations through implementation provides a unique lens

Our deliberate strategy to explore anticipatory conversations in the context of implementation allowed us to have a window in the dynamic and messy process of implementing change that is perceived as counter-normative in academic medical cultures. Existing literature on addressing microaggressions in clinical learning environments suggests that there is a dichotomization and power differential between supervisors and learners, especially when there is a new relationship between a learner and a clinical preceptor [15]. In such contexts, supervisors therefore have a greater degree of responsibility to role model vulnerability and co-create psychological safety [24], however, an opportunity for pre-emptive conversations has also been conceptualized as an opportunity for power sharing where vulnerable learners can role model such conversations in a manner that makes their supervisors more comfortable and vice versa.

Implementing anticipatory conversations in a dynamic working and learning environment can be different from traditional clerkship rotations and clinical placements. For example, our participants noted that if they have existing relationships that have endured over time where they have not felt safe or comfortable, this may hinder their ability to begin to have such conversations. Similarly, if they perceive their organization as being non-responsive or performative in their approach, they may not have the motivation to engage. Medical learners work and learn in complex environments with staff of varying backgrounds, roles, and hierarchical positions within an organization. Our findings suggest that addressing microaggressions for students or residents also requires creating and cultivating environments that are attuned to how power, hierarchy, and resistance may unfold for diverse roles, professions, and disciplines.

### The influence of power asymmetries on addressing microaggressions

Our findings also provide guidance for educators and academic leaders on how to shift cultural norms and promote trust and power sharing within their organizations. Participants were clear that leaders must play a direct role in role modeling change and facilitating implementation. Yet, leaders are also advised to be transparent and vulnerable during the process. During our analysis, we reflected on how participants were focused on the role of individuals with structural power in the organization, rather than empowered with a sense that they have the power to shape interactions in their area of working and/or learning. Yet, our findings highlight that before any members of an academic department can feel safe and comfortable to address microaggressions, they require a sense of trust in their leaders and their organizations. Leaders and organizations in traditionally hierarchical organizations must therefore be attuned to the dichotomization between individuals who hold power and individuals who feel powerless. Leaders are also encouraged to embed discussions about microaggressions into their organizations daily operations and align them with strategic priorities. Our participants suggested breaking this cycle requires role modeling of vulnerability and space to practice conversations that do not feel traditionally natural or comfortable to them.

Anticipatory approaches to fostering belonging or addressing microaggressions have been framed in the medical education literature as a form of intellectual candor that invites vulnerability while building credibility [19]. Although intellectual candor can build trust [25], such candor also requires a mutual form of trust within and between different roles, identities, and hierarchical statutes within an organization. The findings from our participants also suggest that leaders alone cannot foster trust and safety unless there are organizational mechanisms and supports that decrease fear and shift cultural norms for staff who have experienced harm, marginalization, or mistrust from the organizations or leaders who continue to serve within them.

### Limitations

We chose to deliberately explore pre-emptive approaches through the implementation of such approaches in a specific context and setting. Our decision to conduct our study within a Department of Psychiatry is important to consider. In addition, our research team includes someone with a leadership role within the organization of study. We recognize that the researchers’ potential relationships with participants (actual and perceived) represents a potential limitation, however, we established multiple safeguards to ensure that this limitation did not adversely influence the rigor of our work. We attempted to address this through our approach to reflexivity, having a research associate conduct interviews, and de-identifying data for team members who worked in the same organization before analysis. Overall, we believe that there is a need for more research like ours that foster the implementation of evidence-informed approaches in various contexts in medical education to facilitate the translation of medical education research into practice. Another potential limitation to consider as that individuals identifying as women were over-represented in our sample. However, we believe our study adds value because of the nature and complexity of our chosen setting and the rigor with which we approached our data collection and analysis.

## Conclusion

Implementing anticipatory approaches to addressing microaggressions in clinical learning environments holds tremendous potential, however, can be constrained by certain cultural norms. Our study suggests that any attempts to address microaggressions requires attention to the ways in which hierarchical organizations may constrain individual agency. In addition, the self-disclosure of one’s social or perceived identity be counter-normative in a culture that expects individuals to compartmentalize between their personal and professional identities. Findings suggest that the degree to which an individual feels comfortable to participate in sensitive conversations about microaggressions is often related to their existing sense of safety in their workplace, their perception of how responsive or performative their organization’s approach may be, and their perceived power within the organization’s hierarchy. Overall, organizational and academic leaders can play a pivotal role in successful implementation by role modeling vulnerability and integrating discussions into an organization’s daily operations.

## Previous presentations

This project was presented at the American Psychological Association Division 45 Conference in 2023 as a poster presentation.
